# CERKL Reduced PI3P/Autophagy to Promote Pancreatic Cancer

**DOI:** 10.1002/cam4.71402

**Published:** 2025-11-25

**Authors:** Wenying Zeng, Yinhui Yang, Wanlian Li, Jian Pan, Borong Huang, Pengcheng Zhang, Juan Xiao

**Affiliations:** ^1^ Guangxi Key Laboratory of Molecular Medicine in Liver Injury and Repair Affiliated Hospital of Guilin Medical University Guilin Guangxi People's Republic of China; ^2^ Department of Hepatobiliary Surgery Affiliated Shaoyang Central Hospital of University of South China Shaoyang Hunan People's Republic of China; ^3^ Guangxi Health Commission Key Laboratory of Basic Research in Sphingolipid Metabolism Related Diseases Affiliated Hospital of Guilin Medical University Guilin Guangxi People's Republic of China

**Keywords:** autophagy, CERKL, pancreatic cancer, phosphatidyl inositol, Trim21

## Abstract

**Objective:**

Pancreatic cancer (PC) is one of the common malignant tumors in gastrointestinal tract. The roles of CERKL in PC are unknown.

**Methods:**

Here, online databases were used to analyze CERKL mRNA expression and mutation in PC, predict E3 ligase for CERKL. The roles of CERKL were investigated in cells, mice, and clinic samples. The regulations on CERKL by E3 and lipids fluctuations induced by CERKL were analyzed.

**Results:**

It was found that the expression levels of CERKL mRNA and protein were significantly increased in PC. Meanwhile, CERKL promoted PC cells migration and invasion in vitro and in vivo. L296V mutation on CERKL in PC patient was found in cosmic database. Compared with CERKL, CERKL‐L296V could further promote PC cells migration and invasion. Bioinformatics analysis revealed the negative correlation between E3 TRIM21 and CERKL. Furthermore, Trim21 was validated to negatively regulate and bind to CERKL protein. L296V mutation reduced the interaction between CERKL and Trim21, and the ubiquitination on CERKL. Lipidomic analysis showed CERKL down‐regulation could increase phosphatidylinositol amount in PC cells. Phosphatidylinositol addition reversed the effects of CERKL in PC cells. Moreover, CERKL knocked down increased phosphatidylinositol 3‐phosphate (PI3P) content and autophagy. When CERKL was overexpressed, PI3P and autophagy had opposite changes. Of note, CERKL‐L296V had a stronger effect than CERKL on PI3P and autophagy. CERKL induced metastasis could be reduced by autophagy inducer. Thus, CERKL promoted the migration and invasion of pancreatic cancer. L296V mutation enhances the tumor‐promoting effect of CERKL.

**Conclusions:**

TRIM21/CERKL/autophagy pathway exists in PC.

## 1 Introduction

The incidence of pancreatic cancer (PC) is increasing and the mortality rate of PC is high [1]. Surgical resection could be only applied in less than 20% of patients with PC [2]. The pathogenesis of PC is unclear and the effective targets are lack. Thus, PC therapy is unsatisfied.

Ceramide kinase like protein (CERKL) is highly identical to ceramide kinase (CERK) in amino acid sequences [3]. However, the activities of these two enzymes are different. Unlike CERK, the way CERKL regulated cellular sphingolipids including ceramide, 1‐phosphate sphingosine is unknown [4]. Meanwhile, whether CERKL could regulate other lipids is not understood. CERKL could protect cancer cells from oxidative stress and promotes cancer progression [5]. Of note, CERKL gene mutation will lead to its dysfunction, such as failure in cellular oxidative stress response [5]. However, the roles of CERKL in PC remains unclear.

Recently, CERKL has been found to regulate autophagy in neuroblastoma [6]. Deregulated autophagy is closely related to the occurrence and development of PC [7]. Autophagy is identified to degrade organelles through lysosome pathway, playing a crucial role in intracellular environment regulation [8]. Autophagy can not only work against the tumor, but also promote tumor survival under stress conditions [9]. Studies have shown that inhibition of autophagy can promote metastasis of some cancer [10]. However, how CERKL regulates autophagy and the related mechanisms underlying CERKL regulated autophagy in PC remain unclear.

CERKL can be ubiquitinated and degraded through the proteasome pathway [11]. Ubiquitination is one of the most common post‐translational modifications of proteins, which is involved in protein degradation, transport and activation in eukaryotic cells [12]. Ubiquitin protease degradation pathway mainly includes three processes: ubiquitin activator enzyme (E1) bind to and activate ubiquitin molecules, then the activated ubiquitin molecules are transferred to ubiquitin binding enzyme (E2), and finally ubiquitin ligase (E3) mediates ubiquitin transferring to the target proteins [13]. Among them, E3 ligase can specifically bind their substrates and is associated with the progression of cancer [14]. CERKL ubiquitination and related mechanisms in PC are not clear.

In this research, the express pattern of CERKL in PC was firstly described. And then the effects of CERKL and its point mutation in PC cells were investigated. At the same time, the signaling transduction E3/CREKL/autophagy in PC cells were studied.

## 2 Methods and Methods

### 2.1 Chemicals and Antibodies

The following commercial antibodies were used: anti‐CERKL (rabbit, Abcam), anti‐E‐cadherin (rabbit, Abcam), anti‐N‐cadherin (rabbit, Abcam), anti‐Snail (rabbit, Cell Signaling Technology), anti‐Vimentin (rabbit, Cell Signaling), anti‐TRIM21 (rabbit, Proteintech), anti‐P62 (rabbit, Proteintech), anti‐LC3 (rabbit, Sigma), anti‐Ubiquitin (rabbit, Proteintech), anti‐β‐actin (mouse, ZSGB‐BIO), anti‐GAPDH (mouse, ZSGB‐BIO), anti‐GFP (mouse, Origene), anti‐GFP (rabbit, Sangon Biotech), anti‐LaminB1 (rabbit, Cell Signaling Technology), anti‐Tubulin (mouse, Proteintech). (17:0–20:4) PI was from Merck.

### 2.2 Cell Culture

MIA‐Paca2 cells and PANC‐1 cells (China, Shanghai Branch of Chinese Academy of Sciences) were cultured at 37°C with 5% CO_2_ in DMEM (Gibco, 11,330,057) plus 10% FBS, 100 units/mL penicillin and 100 μg/mL streptomycin (Invitrogen, 10,378,016).

### 2.3 Animal Model

Male C57BL/6 mice, 18–20 g, were purchased from Hunan SLAC Laboratory Animal Co. LTD. Panc02 cells stably expressed the control vector or CERKL overexpressing vector were constructed. The mice were divided into the control group and the CERKL overexpression group. The relevant Panc02 stable cells in 0.2 mL (2 × 10^7^ mL^−1^) for each mouse were injected in each group. After tail vein injection, the mice were kept for an additional 28 days. The lungs of the mice were collected after being sacrificed, and H&E staining was performed for pathological observation.

### 2.4 Bioinformatics Analysis

The mRNA expression in PC patients was analyzed on the online database GEPIA2 (http://gepia2.cancer‐pku.cn). The E3 negatively correlated with CERKL was predicted using the LinkedOmics database [15].

### 2.5 Immunohistochemistry (IHC)

After dewaxing, hydration and antigen retrieval, PAAD tissue microarray (Shanghai Core Ultra Biological Co. Ltd., HPanA020PG01), was incubated with anti‐CERKL antibody or LC3 antibody. Finally, the CERKL protein intensity (H‐score) in each sample was analyzed by the HALO digital pathological image analysis platform. LC3II expression was analyzed by the pathologist.

### 2.6 Western Blotting

Proteins in cell lysate were separated by SDS–PAGE and transferred to PVDF membranes (Millipore). Membranes loaded with proteins were incubated with indicated primary antibodies and then with HRP‐conjugated secondary antibodies; the indicated proteins' intensity was visualized using ECL substrates.

### 2.7 Transwell Assay

After indicated treatments, PC cells (10^5^/mL) were cultured in serum‐free medium in the upper trans‐well chamber with or without matrigel. The lower chambers were loaded with complete culture medium. 48 h later, cells in the transwell were fixed with 4% PFA followed by crystal violet staining. Migrative or invasive cells were imaged and cells in the picture were calculated with ImageJ software.

### 2.8 Cytoplasm and Nucleus Fractions Purification

PC cells (Panc‐1) were transfected with GFP‐CERKL or GFP‐CERKL L296V expression vectors. 48 h later, cells were digested and treated with Nuclear and Cytoplasmic Protein Extraction Kit (Beyotime Biotechnology) according to the instructions. LaminB1 and tubulin antibodies were used to identify nucleus and cytoplasm fractions.

### 2.9 Lipidomics Assay

PC cells (MIA) were transfected with control shRNA or shRNA targeting CERKL. 48 h later, equal amounts of cells (≈10^6^) were collected followed by liquid nitrogen flash freezing. Lipidomics assay was performed by SHANGHAI BIOTREE BIOMEDICAL TECHNOLOGY CO. LTD.

### 2.10 Binding or Ubiquitination Assay

Binding assay, 293 T cells were transfected with control vector, GFP‐CERKL expressing vector or GFP‐CERKL‐L296V expressing vector for 48 h. Cells were lysed with RIPA buffer (Beyotime Biotechnology) with protease/phosphatase inhibitor cocktails. Cell lysate was incubated with anti‐GFP antibody then with Protein G Magnetic Beads (Thermo). The proteins enriched on the beads were lysed with 5× SDS‐PAGE loading buffer and analyzed by WB with indicated antibodies.

Ubiquitination assays were done following a denaturing IP protocol. Indicated plasmids were transfected into Panc‐1 cells for 48 h. Then the cells were lysed in lysis buffer containing 1% SDS and denatured by 95°C heating for 10 min. The samples were centrifuged at 12,000 *g* for 10 min, and then the supernatants were diluted with lysis buffer until the SDS concentration was decreased to 0.1% for IP with anti‐GFP antibody (Sangon Biotech, Shanghai).

### 2.11 PI3P Assay

PC cells (MIA) were transfected with indicated plasmids for 48 h. Equal amounts of cells were collected. Cellular PI3P was analyzed by ELISA kit (Echelon) according to the protocol provided by the manufacturer.

### 2.12 Immunostaining

Cells under indicated treatments were fixed with 4% paraformaldehyde and then penetrated with 0.1% Triton X‐100. The cells were blocked with 5% goat serum. Anti‐LC3 antibody or trim21 antibody was added to the cells at 37°C for 1 h. Goat polyclonal secondary antibody to rabbit (Alexa Fluor 647) (abcam) was added to the cells at room temperature for 1 h. Signaling was observed by fluorescence microscopy.

### 2.13 Statistical Analysis

The standard deviation is based on the mean ± SD of three independent experiments. Student *t* test or One‐way Analysis of Variance was used as statistical analysis by using SPSS. *p < 0.05, **p < 0.01, ***p < 0.001 and **** *p* < 0.0001.

## 3 Results

### 3.1 CERKL Increased in PC

**FIGURE 1:**
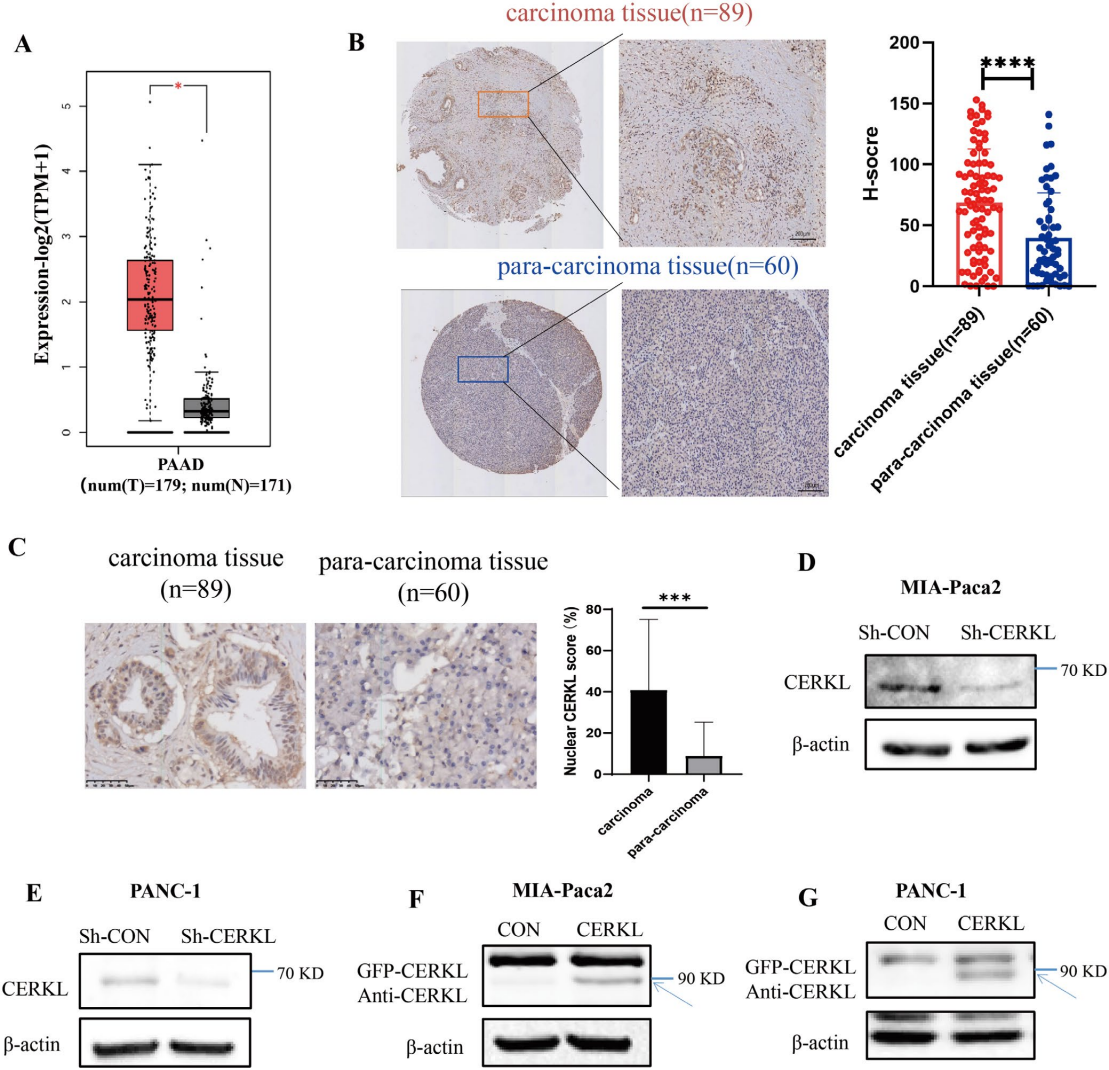
CERKL increased in PC. The mRNA expression of CERKL was analyzed on online database GEPIA2 (http://gepia2.cancer‐pku.cn) (A). The protein expression of CERKL was analyzed on PC tissue array using IHC with anti‐CERKL antibody, the protein intensity (H‐score) of each clinic sample was evaluated by HALO digital pathological image analysis platform and was present in graph, H‐score difference in two groups was analyzed with student *t* test (****p < 0.0001) (B). The ratios of nuclear CERKL were analyzed and the difference of the ratios between two groups was calculated with student *t* test (***p < 0.001) (C). (D and E) Control shRNA or shRNA targeting CERKL were transfected into indicated PC cells. (F and G) Control vector or CERKL expressing vector were transfected into indicated PC cells. 48 h later, cells were lysed and analyzed by WB with anti‐CERKL antibody.

Firstly, the expression levels of CERKL mRNA in PC tissues were significantly higher than that in normal pancreas tissues (*p* < 0.05) according to online TCGA gene tablature and survival analysis GEPIA2 (http://gepia2.cancer‐pku.cn) (Figure 1A). In order to validate the bioinformatics analysis result, immunohistochemistry was performed using a tissue array (cancer *n* = 89, adjacent noncancerous tissue *n* = 60). CERKL signaling in cancer and paracancer tissues was found in both the nucleus and cytoplasm (Figure 1B). Statistical results showed that the expressions of CERKL protein in PC tissues were significantly higher than that in paracancer tissues (*p* < 0.0001) (Figure 1B). Meanwhile, it could be found that the percentages of nuclear CERKL in PC tissues were significantly larger than that in the paracancer tissues (Figure 1C).

### 3.2 CERKL Promoted the Migration and Invasion of PC Cells

To investigate the roles of CERKL in PC, we firstly checked the transfection efficiency of the related plasmids. PC cells were transfected with Control/CERKL shRNA or Control/GFP‐CERKL expressing vector. It was found that CERKL knockdown or overexpression was good (Figure 1D–1G).

**FIGURE 2:**
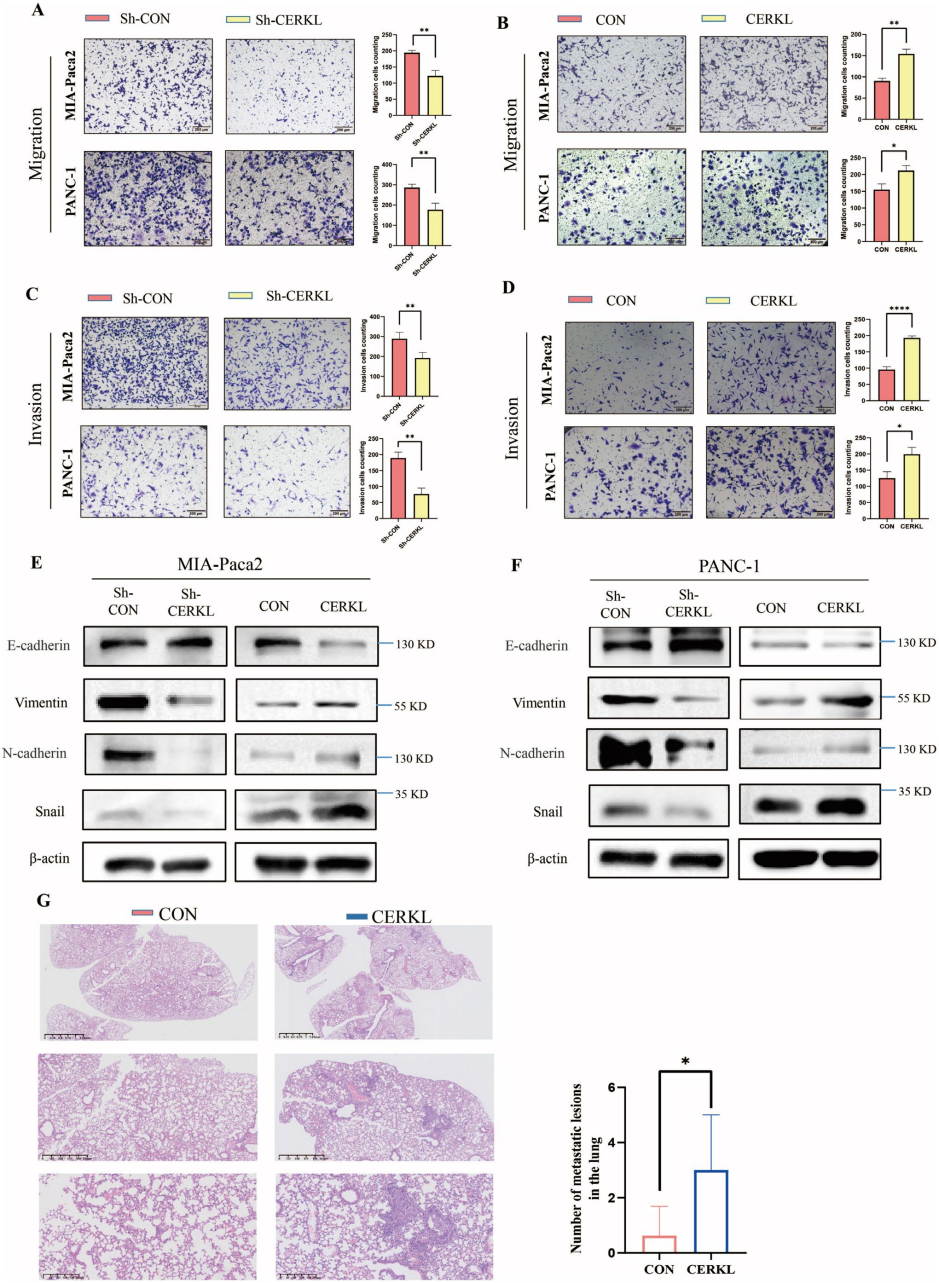
CERKL promoted PC cells migration and invasion. Control shRNA (vector) or shRNA targeting CERKL (CERKL expressing vector) were transfected into indicated PC cells. 48 h later, cells under indicated treatments were subjected to transwell assay (A–D) or WB (E and F), migration (A and B) or invasion (C and D) cells in indicated groups were counted using ImageJ and represented as mean ± SD. Cell numbers difference in two groups were analyzed with student *t* test (*p < 0.05, **p < 0.01, ****p < 0.0001). (G) PC cells stable expressing with control vector or CERKL expressing vector were injected into mice. 3 weeks later, lung tissues were collected and subjected to HE staining for pathological analysis. Metastatic lesions nubmers in the lung were counts, difference in two groups were analyzed with student *t* test (*p < 0.05).

The effect of CERKL on PC cells proliferation was then investigated. However, whether CERKL was knocked down/overexpressed or not, PC cells growth did not change (Figure S1). The ability of migration and invasion in PC cells representing metastasis were analyzed. When CERKL was knocked down, PC cells migration and invasion were inhibited (Figure 2A,C). Similar results were got when another shCERKL plasmid was used (Figure S2A–E). While, Overexpression of CERKL significantly enhanced the migration and invasion of PC cells (Figure 2B,D). Meanwhile, EMT markers regulated by CERKL were also detected. Knockdown of CERKL in PC cells significantly increased the expression of epithelial cell markers E‐cadherin and decreased the expression of mesenchymal cell markers including N‐cadherin, vimentin and snail (Figure 2E,F, Figure S2F). The results were reversed after CERKL overexpression (Figure 2E,F). The results suggested that CERKL promoted PC cells migration and invasion.

### 3.3 CERKL Promotes PC Cells Metastasis In Vivo

As can be seen from the above, CEREL can promote the ability of PC cells' migration and invasion in vitro. To further verify the effect of CERKL on PC, we constructed a C57BL/6 mouse metastasis model to observe the effect of CERKL on PC in vivo. Mice were intravenously injected with mouse PC cells stably expressed with control or CERKL‐expressing vector. The results showed that, compared to the control group, more mice in the CERKL overexpression group showed more lung metastases (Figure 2G), suggesting that CERKL promoted the migration and invasion of pancreatic cancer cells in vivo.

### 3.4 CERKL Point Mutation L296V Further Promoted PC Cells Migration and Invasion

**FIGURE 3:**
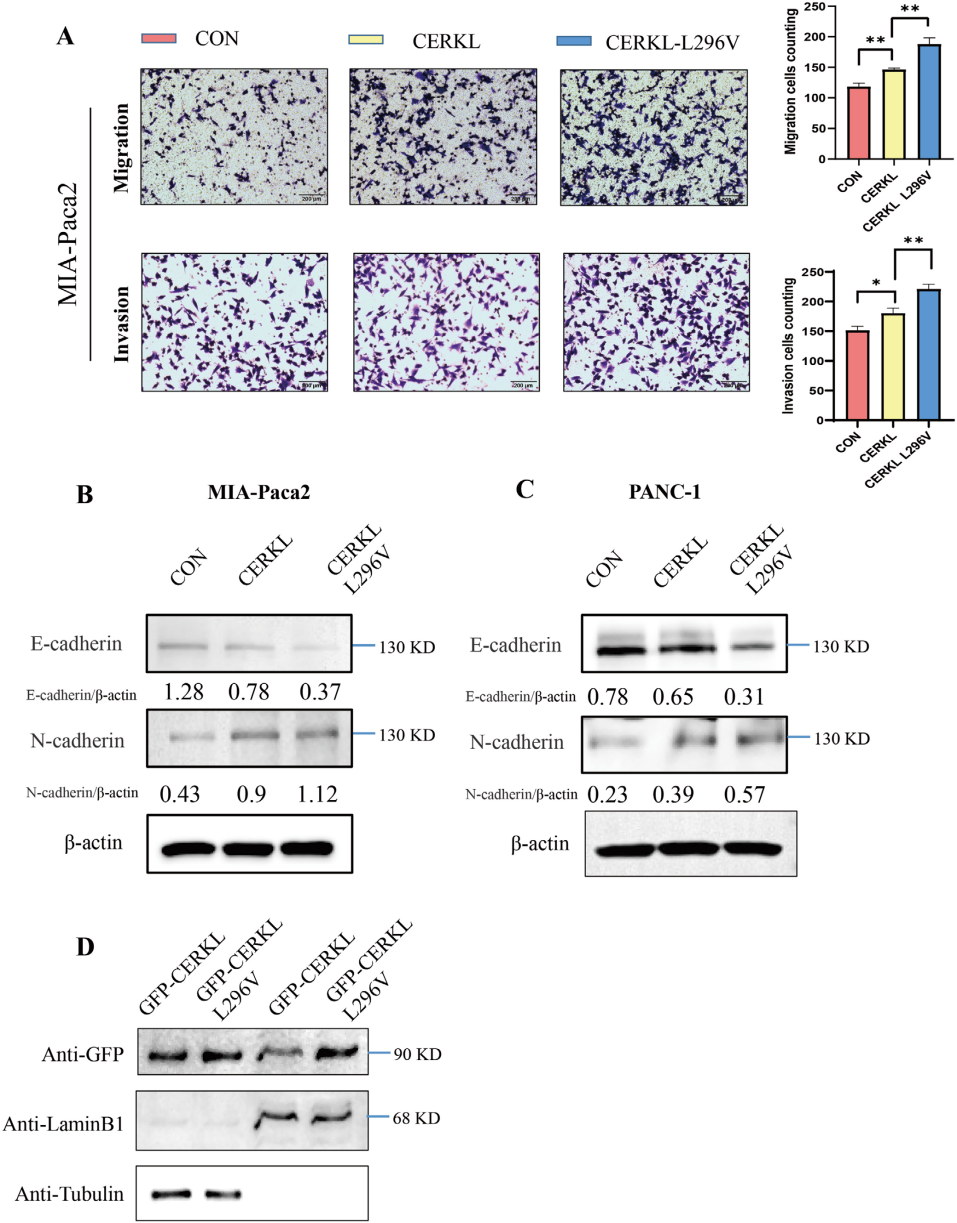
L296V mutation strengthened CERKL function. PC cells were transfected with control vector, CERKL expressing vector or CERKL‐L296V expressing vector. 48 h later, cells under indicated treatments were subjected to transwell assay (A), WB (B and C) or cytoplasm and nucleus fractions purification (D), migration or invasion cells in indicated groups were counted using ImageJ and represented as mean ± SD. Cell numbers difference in three groups were analyzed with ANOVA (*p < 0.05, **p < 0.01). Proteins intensity was qualified using ImageJ software.

COSMIC database revealed the presence of CERKL‐L296V mutants in PC patients. To investigate whether the mutation affected CERKL function, the CERKL‐L296V plasmid was constructed. The number of cells transfected with CERKL‐L296V passing through the trans‐well chamber was significantly increased compared with CERKL (Figure 3A). Consistent with the trans‐well assay, the expression of the mesenchymal cell marker (N‐cadherin) of CERKL‐L296V was increased compared with CERKL, while the epithelial cell marker (E‐cadherin) expression was decreased compared with CERKL (Figure 3B,C). These results suggested that the CERKL‐L296V point mutation could further promote PC cells migration and invasion. Moreover, more CERKL‐L296V was located in the nuclear than the wild type (Figure 3D). It was indicated that CERKL function was changed after the point mutation, and leucine 296 on CERKL might be an important site for the PC cells migration and invasion regulated by CERKL. Taken together, CERKL translocation from cytoplasm to nucleus might be required for its function in PC.

### 3.5 CERKL Was Negatively Regulated by TRIM21

**FIGURE 4:**
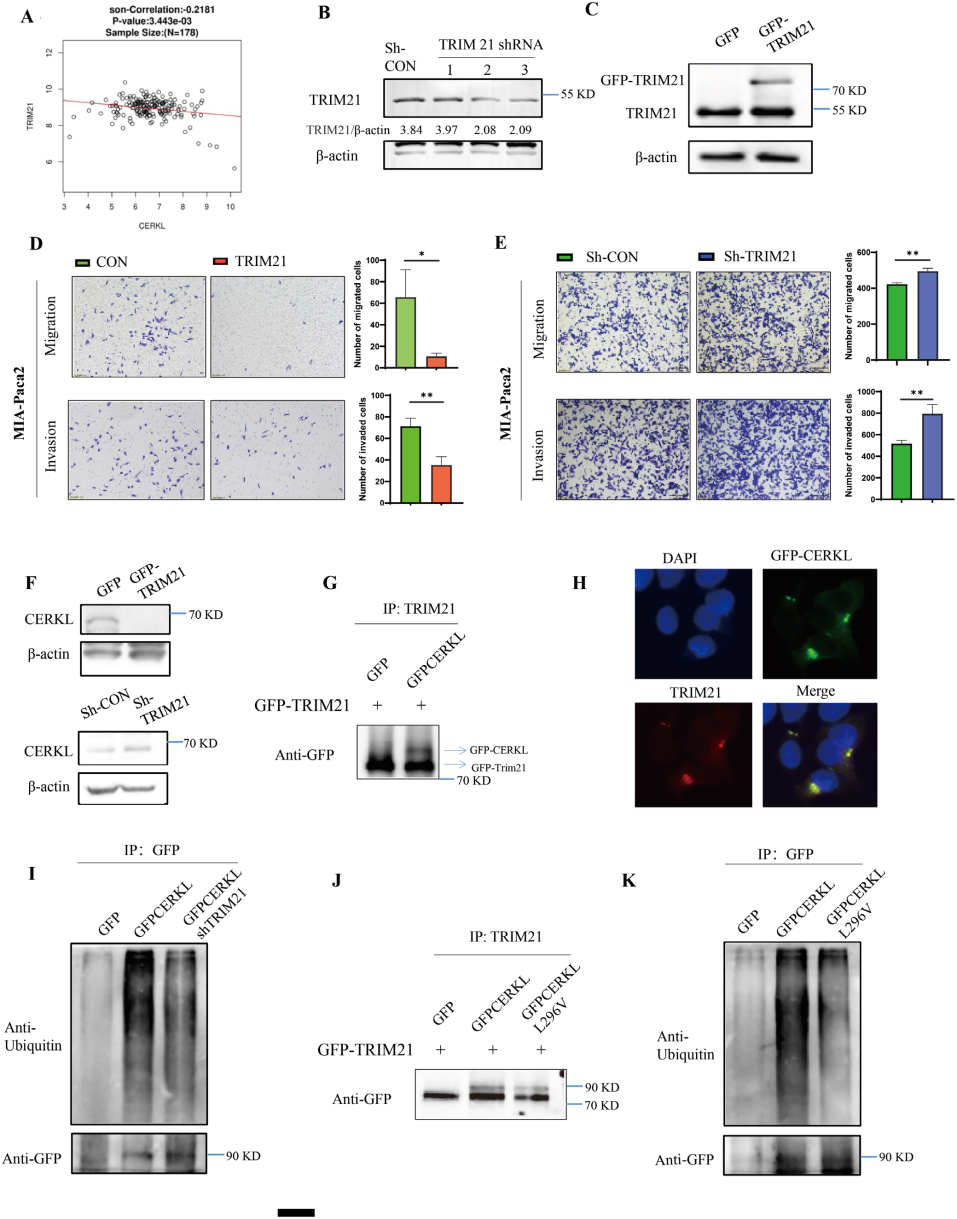
L296 was important for CERKL interaction regulation by TRIM21. (A) The TRIM21 negatively correlated to CERKL was predicted using LinkedOmics database. (B–E) Control shRNA (vector) or shRNA targeting TRIM21 (TRIM21 expressing vector) were transfected into indicated PC cells. 48 h later, cells under indicated treatments were subjected to WB (B and C) or transwell assay (D and E), migration or invasion cells in indicated groups were counted using ImageJ and represented as mean ± SD. Cell numbers difference in two groups were analyzed with student *t* test (*p < 0.05, **p < 0.01). Proteins intensity was qualified using ImageJ software. (F) PC cells were transfected with indicated plasmids and then cells were lysed, cell lysate were subjected to WB. (G, I–K) 293 T cells (G and J) PC cells (I and K) were transfected with indicated plasmids, cells were lysed with IP buffer, cell lysate were incubated with anti‐TRIM21 (G, J) or anti‐GFP (I, K) antibody, after enrichment with Protein G Magnetic Beads, proteins on the beads were analyzed with WB. (H) Cells under indicated treatments were fixed and sent for immunofluorescence assay with anti‐TRIM21 antibody, GFP fluorescence (GFP‐CERKL), red fluorescence (TRIM21) were imaged.

CERKL was increased in PC, but its upstream regulator was unknown. Bioinformatics analysis showed that there was a negative correlation between E3 ligase TRIM21 and CERKL (Figure 4A). Next, the roles of TRIM21 in PC cell migration and invasion were investigated. TRIM21 knockdown promoted PC cell migration and invasion, while TRIM21 overexpression inhibited PC cell migration and invasion (Figure 4B–4E). These results indicated that TRIM21 inhibited PC. The relationship between TRIM21 and CERKL was further validated, and it was found that the high expression of TRIM21 reduced the level of CERKL protein, while the low expression of TRIM21 led to the increase of CERKL expression (Figure 4F). As the E3, TRIM21 might bind to CERKL to regulate its protein expression. Thus, we investigated the interaction between CERKL and TRIM21. As we expected, CERKL was also detected in the immunoprecipitation component obtained with anti‐TRIM21 antibody (Figure 4G). Moreover, we detected the co‐localization of TRIM21 and CERKL. The green fluorescence of CERKL was found to be co‐localized with the red fluorescence of TRIM21 (Figure 4H). TRIM21 could induce ubiquitination on its substrates. Considering the regulation of CERKL by TRIM21, we conducted a ubiquitination assay. When TRIM21 was knocked down, the ubiquitination was reduced (Figure 4I). CERKL and CERKL‐L296V were enriched respectively in the immunoprecipitation experiment. TRIM21 was present as the binding partner, and the mutation of L296V weakened the interaction between the two proteins (Figure 4J). It was found that ubiquitination modification was available on CERKL, and the ubiquitination level of CERKL‐L296V was reduced (Figure 4K). These results suggested that leucine 296 might be an important site for CERKL regulation by TRIM21.

### 3.6 CERKL Regulated PC Cells Migration and Invasion Through Phosphatidylinositol

**FIGURE 5:**
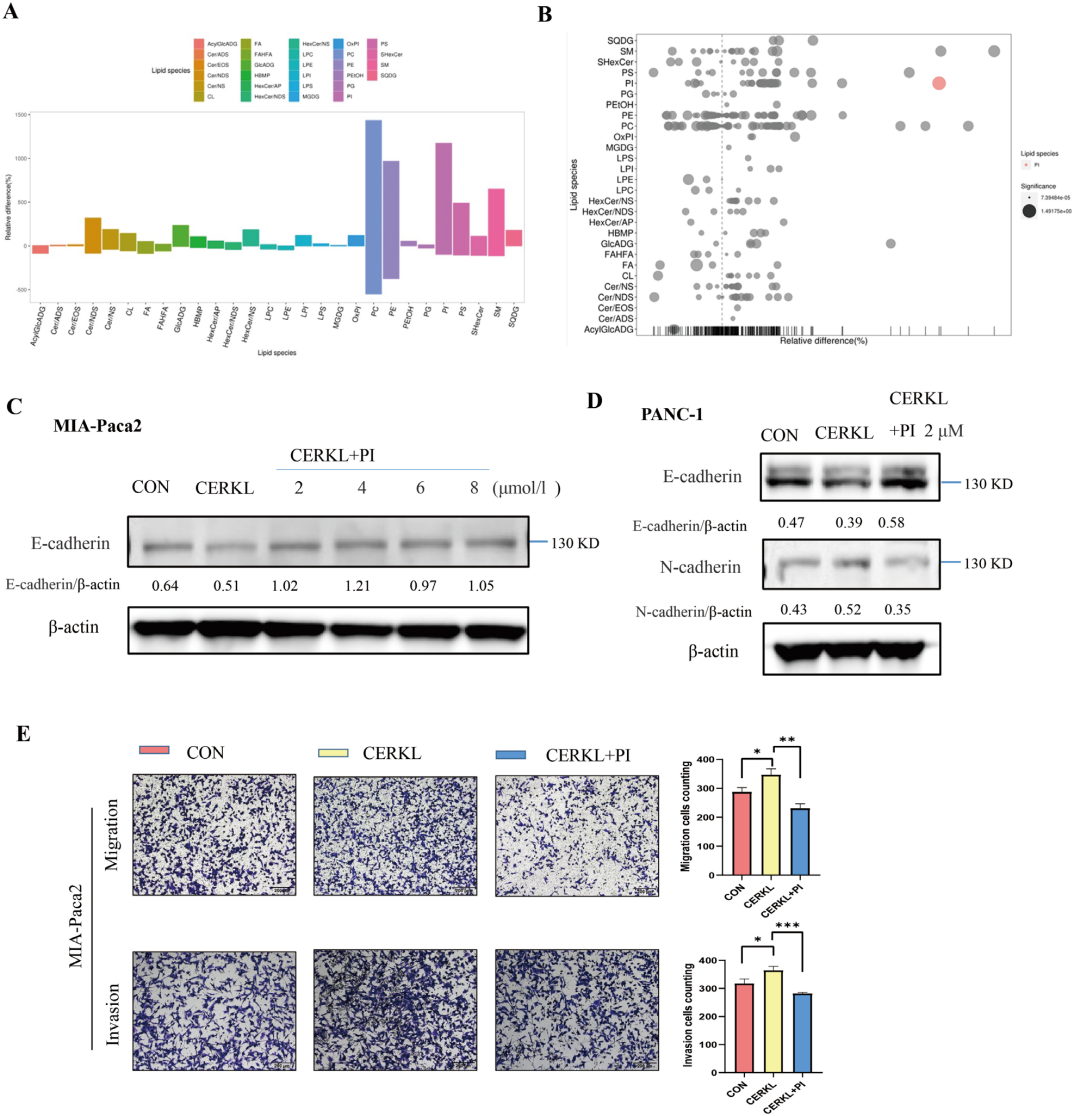
CERKL worked on PC cells migration as well as invasion through PI. (A, B) PC cells were transfected with control shRNA or shRNA targeting CERKL and then cells were sent for lipidomics assay, the difference of each type of lipid in CERKL knockdown PC cells compared with control was visualized in bar plot (A) or bubble plot (B), positive relative difference ratio represented increment (A) and color bubble represented significance (B). (C–E) Cells under indicated treatments were subjected to WB (C and D) or transwell assay (E), migration or invasion cells in indicated groups were counted using ImageJ and represented as mean ± SD. Cell numbers difference in three groups were analyzed with ANOVA (*p < 0.05, **p < 0.01, ***p < 0.001). Proteins intensity was qualified using ImageJ software.

These above results indicated that CERKL promoted PC cells' migration and invasion in vitro and in vivo, and it was regulated by TRIM21. CERKL was reported to affect cellular sphingolipid. To investigate whether the effect of CERKL is related to lipid changes, non‐targeted lipidomics analysis was performed. Results showed that inhibition of CERKL expression in PC cells resulted in a statistically significant increase in phosphatidyl inositol (PI) content but not in sphingolipids (Figure 5A,B). Therefore, we speculated that PI might contribute to PC cells' migration and invasion inhibition caused by CERKL down‐regulation. To verify this hypothesis, we added exogenous PI. It was found that PI reversed E‐cadherin and N‐cadherin protein expression exerted by CERKL (Figure 5C,D). Meanwhile, PI reduced the PC cells' migration and invasion induced by CERKL (Figure 5E).

### 3.7 CERKL Inhibits PI3P‐Autophagy

**FIGURE 6:**
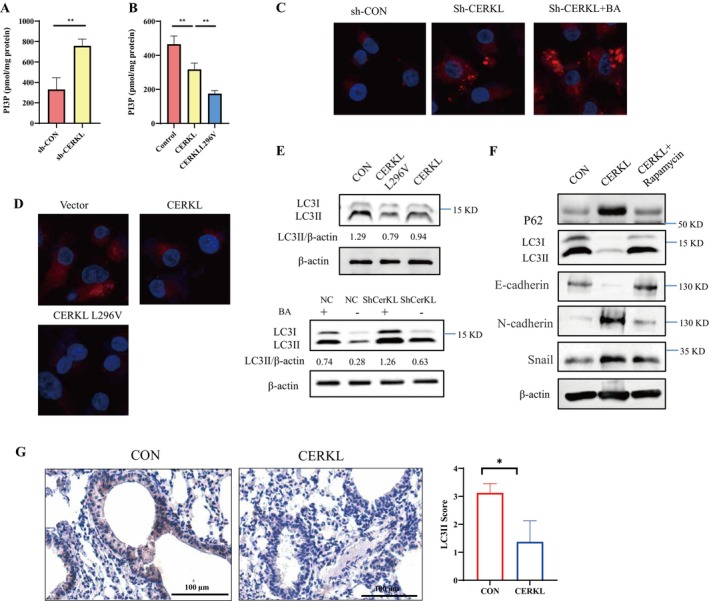
CERKL reduced PI3P‐autophagy. PC cells were transfected with indicated plasmids, 48 h later, bafilomycin A1 (BA) was added for additional 3 h (C–E), cells were lysed (A, B, E and F) or fixed (C, D). Cells lysate were sent for ELISA (A and B) or wb (E and F), differences of PI3P amounts in different groups were analyzed with student *t* test (A) or ANOVA (**p < 0.01) (B), proteins intensity was qualified using ImageJ software (E and F). Cells were sent for immofluorescence assay with anti‐LC3 antibody (red fluorescence, C and D). (G) PC cells stable expressing with control vector or CERKL expressing vector were injected into mice. 3 weeks later, lung tissues were collected and subjected to IHC staining and LC3II score was evaluated, differences between two groups were analyzed with student *t* test (*p < 0.05).

CERKL affects the amount of PI in PC cells. PI can be converted into phosphatidylinositol 3‐phosphate (PI3P). Therefore, we detected the level of PI3P by ELISA and found that knocking down CERKL did increase the level of PI3P (Figure 6A). The level of PI3P decreased significantly after transfection of the overexpression plasmid CERKL and the point mutant CERKL‐L296V in PC cells, and the level of PI3P decreased further under the overexpression of the point mutant CERKL‐L296V than that of CERKL (Figure 6B). PI3P as the component of autophagosome, directly regulates autophagy [16, 17, 18]. Thus, CERKL might work on autophagy. Immunofluorescence results showed that the expression of autophagosome‐anchored LC3II was enhanced after CERKL knockdown (Figure 6C). Bafilomycin A1, an autophagic flux inhibitor, was used to investigate whether CERKL knockdown truly promoted autophagy. CERKL knockdown increased LC3II intensity further in the presence of bafilomycin A1 (Figure 6C). Of note, while the LC3II was weakened after overexpression of CERKL in PC cells (Figure 6D). CERKL L296V decreased LC3 intensity further (Figure 6D). The WB readouts of LC3II protein were consistent with the immunofluorescence results (Figure 6E, Figure S2G). Finally, EMT markers were investigated. It was found that autophagy increment reversed E‐cadherin, N‐cadherin and snail expression exerted by CERKL (Figure 6F). Moreover, LC3 II was reduced in the lung metastasis with overexpression of CERKL (Figure 6G). Combined with the above results, it was suggested that CERKL could affect PI3P‐autophagy and that it regulated PC cells metastasis might be through autophagy.

## 4 Discussion

In this research, CERKL mRNA and protein expression were found to significantly increase in PC. Meanwhile, CERKL promoted the migration and invasion of PC cells. The L296V mutation on CERKL present in a PC patient was found, and CERKL‐L296V could further promote the migration and invasion of PC cells. Furthermore, predicting TRIM21 was validated to negatively regulate and bind to CERKL protein. The L296V mutation reduced the interaction between CERKL and TRIM21, and the ubiquitination on CERKL. CERKL down‐regulation could increase PI amount in PC cells. PI addition reversed the effects of CERKL in PC cells. Moreover, CERKL knocked down increased PI3P content and autophagy. When CERKL was overexpressed, PI3P and autophagy had opposite changes. Of note, CERKL‐L296V had a stronger effect than CERKL on PI3P and autophagy. Trim21 worked contrary to PC. Autophagy activation reduced PC cells' metastasis induced by CERKL.

In this research, CERKL was revealed as the promoter for PC which was consistent with the previous results [4]. Possessing the nuclear localization signal, CERKL is present in nuclear and other organelles. Mutations could cause the redistributions of CERKL. CERKL‐R105A and R257X were found to localize mainly in the nuclear, and their functions were changed [3, 19]. Thus, subcellular location was very important for its physiological and pathological characters. L296V mutations strengthened the tumor‐promoting ability of CERKL. However, whether L296V caused the re‐distribution of CERKL was unknown, which needed to be investigated further. We predicted the ubiquitination site on CST Phosphosite, and K340 on CERKL was the predicted site. L296 is close to K340. Therefore, the L296V mutation on CERKL might probably affect the ubiquitination of CERKL. More CERKL L296V presence in PC cell nuclear might also be due to the decreased ubiquitination.

In the bioinformatics analysis, TRIM21 was found to negatively correlate with CERKL. The TRIM family is identified as the ring finger E3 ligase and their roles in cancers are dual [20, 21]. Some TRIM proteins could promote EMT in cancer through regulating various tumor repressors [22]. Some TRIM proteins could induce the ubiquitination of EMT markers to reverse EMT in PC [23]. The substrates of TRIM21 included cytoplasmic and nuclear proteins [24], and its roles in other cancers had been studied [25]. Previously, our research found that TRIM21 could inhibit PC in vitro and in vivo (not published). Here, this research suggested that TRIM21 inhibited PC through negatively regulating CERKL.

CERKL induces autophagy in ARPE‐19 cells [26] which was in contrast to the results in our study. Thus, the effects of CERKL on autophagy might be cell‐dependent. Autophagy is very important for cellular homeostasis and under some conditions, autophagy can inhibit tumors. Consistently, oncogenes like Akt, RAS and ERK could reduce autophagy by activating mTOR [27, 28]. In our study, CERKL was found to reduce PI and PI3P content; the latter was considered to be necessary for autophagosome formation [29]. Meanwhile, CERKL caused the down‐regulation of LC3II which is located on the elongated autophagosome membrane and was identified as the autophagy maker [30]. Moreover, CERKL down‐regulation decreased p62 which labels the substrates for autophagy degradation and also works as the autophagy marker [31]. LC3II decrement indicated autophagy inhibition. LC3II increment and p62 decrement represented autophagy flux up‐regulation. Therefore, CERKL inhibited PI3P‐autophagy.

In this research, we found that PI3P was reduced when CERKL was overexpressed and opposite results were obtained in the case of CERKL knockdown. The effect of CERKL on LC3II was consistent with that on PI3P. Therefore, we think CERKL regulated autophagy through PI3P. Further, we found that, snail which works as the transcriptor for EMT‐promoting proteins, was positively regulated by CERKL. Moreover, the autophagy inducer could reduce snail in the case of CERKL overexpression. Thus, we think autophagy could reduce the protein level of snail and this might be due to the autophagic degradation. CERKL regulated autophagy to affect the snail following PC migration. Of note, CERKL translocation into the nuclear might be important for autophagy regulation.

CERKL could protect cancer cells from oxidative stress to maintain cellular homeostasis [32]. CERKL down‐regulation led to reactive oxygen species (ROS) production. ROS could induce autophagy. Thus, CERKL inducing autophagy might be through ROS.

Previous research [33] showed that phosphatidic acid could be transformed into diacylglycerol (DAG) by phosphatidic acid phosphatase. Phosphatidic acid could also be transformed into cytidinediphosphatediacylglycerol (CDP‐DAG) by CDP‐DAG synthase. CDP‐DAG could be hydrolyzed into phosphatidylglycerol and PI. Our study found that CERKL induced the protein expression of phosphatidic acid phosphatase 2c (data not shown). Thus, the regulation of PI production by CERKL might be through the competitive production of DAG and CDP‐DAG; this hypothesis needs to be investigated.

## 5 Conclusions

In conclusion, CERKL promoted the migration and invasion of pancreatic cancer. L296V mutation enhances the tumor‐promoting effect of CERKL. TRIM21/CERKL/autophagy pathway exists in PC.

## Author Contributions

**Wenying Zeng:** writing – original draft, investigation, visualization. **Yinhui Yang:** writing – original draft, investigation. **Wanlian Li:** writing – original draft, investigation. **Jian Pan:** writing – original draft, investigation. **Borong Huang:** investigation, writing – original draft. **Pengcheng Zhang:** writing – original draft, investigation. **Juan Xiao:** writing – original draft, funding acquisition, writing – review and editing, project administration.

## Disclosure

The authors declare that none of the AI and AI‐assisted technologies was used.

## Ethics Statement

All animal care and experimental procedures were approved by the Ethical Committee on Animal Experiments at Guilin Medical University (approved no. GLMC201803030) and following the National Institutes of Health guide for the care and use of laboratory animals (NIH Publications no. 8023, revised 1978).

## Consent

The authors have nothing to report.

## Conflicts of Interest

The authors declare no conflicts of interest.

## Supporting information


**Figure S1:** CERKL didn't affect cell growth. Control shRNA (vector) or shRNA targeting CERKL (CERKL expressing vector) were transfected into indicated PC cells. 48 h later, cells under indicated treatments were subjected to CCK‐8 assay.

**Figure S2:** CERKL knockdown using another shRNA plasmid inhibited PC cells migration and invasion, induced autophagy. Control shRNA or another shRNA targeting CERKL were transfected into panc‐1 (A–C, F and G) or mia‐paca2 (D–F) cells. 48 h later, cells under indicated treatments were subjected to transwell assay (A–E) or WB (F and G), migration cells were tested using RTCA instrument (A), migration (D) or invasion (C and E) cells in indicated groups were counted using ImageJ and represented as mean ± SD. Cell numbers difference in two groups were analyzed with student *t* test (* *p* < 0.05, ** *p* < 0.01).

## Data Availability

http://gepia2.cancer‐pku.cn online database, LinkedOmics database were used. The datasets generated during and/or analyzed during the current study are available from the corresponding author on reasonable request.
