# Supplementary figures and images for: CERKL Reduced PI3P/Autophagy to Promote Pancreatic Cancer
Source: Cancer Med. 2025 Nov 25;14(22):e71402. doi: 10.1002/cam4.71402 (PMC12645229; doi:10.1002/cam4.71402)

# Figure. S1

**A****MIA-Paca2**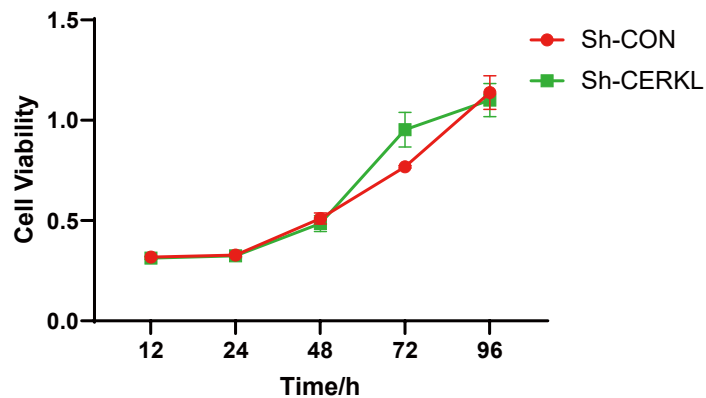**B****PANC-1**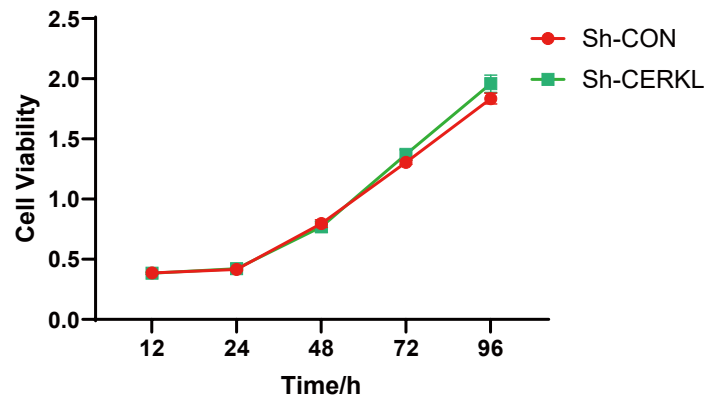**C****MIA-Paca2**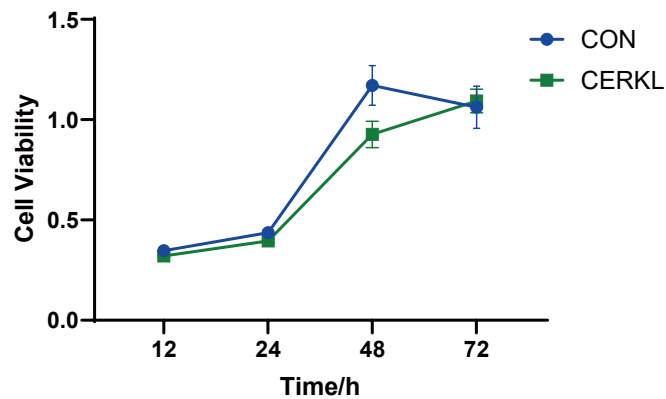**D****PANC-1**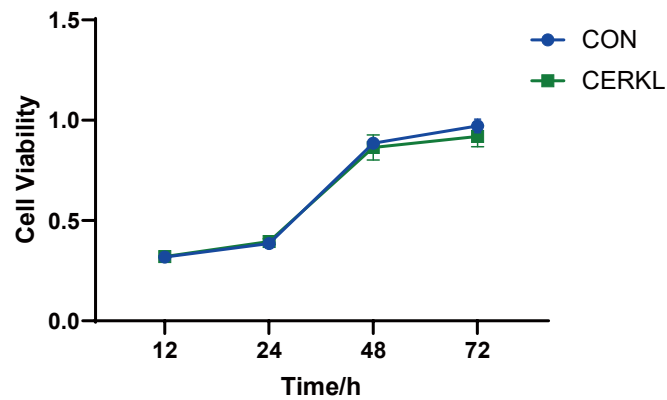

Supplement: Supplementary file 1 — Figure S1: CERKL didn't affect cell growth. Control shRNA (vector) or shRNA targeting CERKL (CERKL expressing vector) were transfected into indicated PC cells. 48 h later, cells under indicated treatments were subjected to CCK‐8 assay. [file CAM4-14-e71402-s002.pdf]

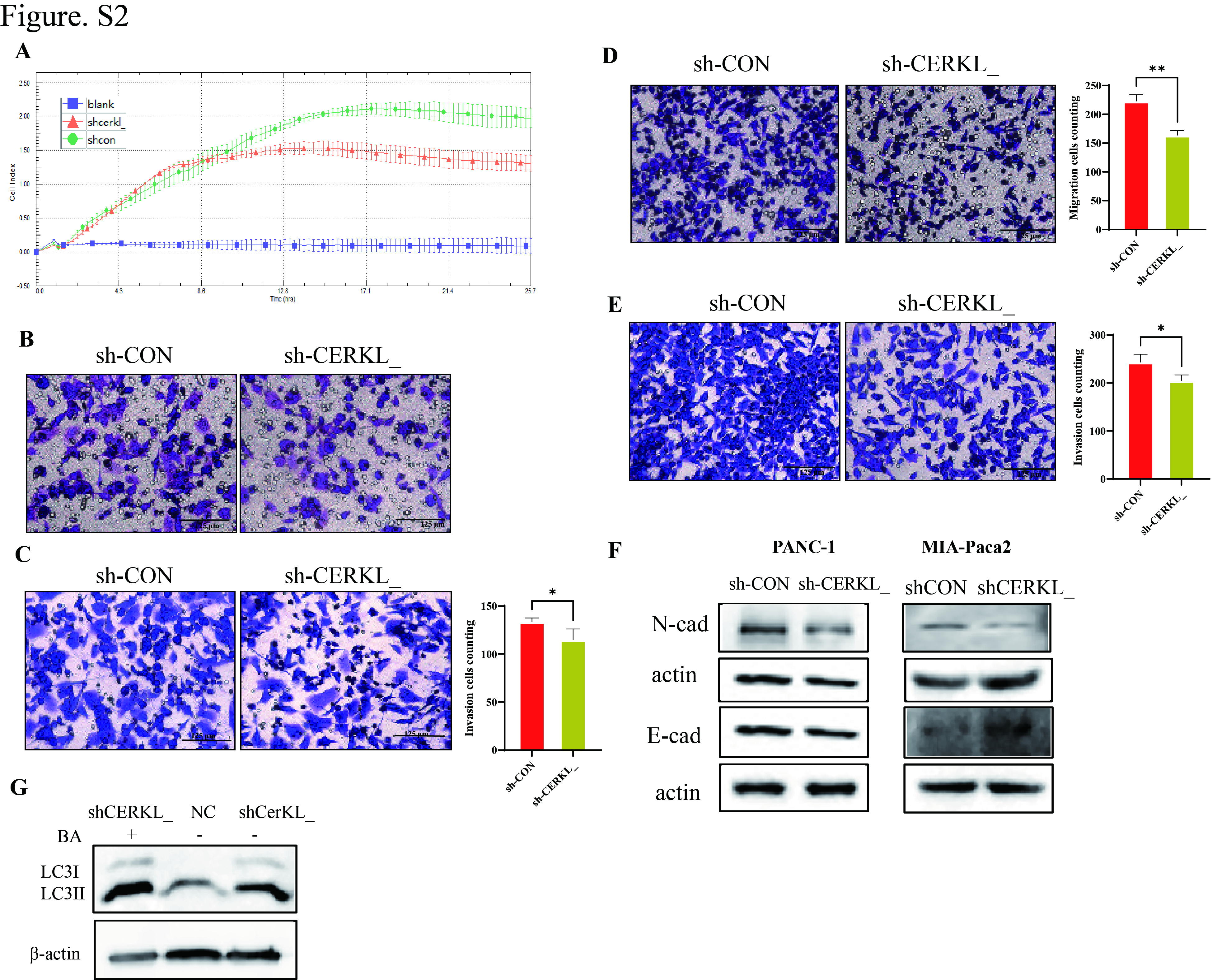

Supplement: Supplementary file 2 — Figure S2: CERKL knockdown using another shRNA plasmid inhibited PC cells migration and invasion, induced autophagy. Control shRNA or another shRNA targeting CERKL were transfected into panc‐1 (A–C, F and G) or mia‐paca2 (D–F) cells. 48 h later, cells under indicated treatments were subjected to transwell assay (A–E) or WB (F and G), migration cells were tested using RTCA instrument (A), migration (D) or invasion (C and E) cells in indicated groups were counted using ImageJ and represented as mean ± SD. Cell numbers difference in two groups were analyzed with student t test (* p < 0.05, ** p < 0.01). [file CAM4-14-e71402-s001.jpg]
